# Comparison and Agreement of Criteria for Classifying Improvement in Walking Ability During Inpatient Rehabilitation After Spinal Cord Injury

**DOI:** 10.3390/healthcare14162668

**Published:** 2026-08-21

**Authors:** Urška Puh, Tea Čuk, Bojan Čeru, Gaj Vidmar

**Affiliations:** 1 University Rehabilitation Institute, 1000 Ljubljana, Slovenia; tea.cuk@ir-rs.si (T.Č.); bojan.ceru@ir-rs.si (B.Č.); gaj.vidmar@ir-rs.si (G.V.); 2 Faculty of Health Sciences, University of Ljubljana, 1000 Ljubljana, Slovenia; 3 Faculty of Medicine, University of Ljubljana, 1000 Ljubljana, Slovenia; 4 Faculty of Mathematics, Natural Sciences and Information Technologies, University of Primorska, 6000 Koper, Slovenia

**Keywords:** spinal cord injury, walking capacity, 6MWT, 10MWT, walking performance

## Abstract

**Background/Objectives:** To evaluate classification of improvement and agreement of walking-ability improvement criteria derived from outcome measures of walking capacity and performance among inpatients with spinal cord injury (SCI). **Methods**: Retrospective analysis of admission and discharge data from adults with SCI in the single-centre database from years 2020 to 2023: Six-minute Walk Test (6MWT), Ten-metre Walk Test (10MWT), Walking Index for Spinal Cord Injury; selected items of Spinal Cord Independence Measure (SCIM-III), and Functional Independence Measure; Spinal Cord Injury Functional Ambulation Inventory (SCI-FAI) Walking Mobility subscale. Twenty-three criteria for assessing walking-ability improvement including distribution-based change threshold (DBCT) and provisional milestones for in-home or in-the-community walking (derived from stroke population) and/or related independence were evaluated. Proportion of patients who progressed according to each criterion and agreement between criteria were calculated. **Results:** Data from 281 individuals were analysed (median 2 months since SCI). Improvement in SCIM-III Mobility Categories was achieved in 47%. Estimated DBCT of 6MWT (69 m) was exceeded in 46%, and DBCT of 10MWT at comfortable speed (CS) in 42% of participants. Forty-two percent of participants improved in provisional Walking Ability Categories (WACs) based on 6MWT or 10MWT_CS, 39% on SCIM-III-Stairs criterion, and 36% on the SCI-FAI criterion. **Conclusions:** The estimated DBCT and the WACs for 6MWT and 10MWT_CS are promising criteria to assess improvement in walking capacity after rehabilitation in patients with SCI; however, they require prospective validation against patient-centred and real-world mobility anchors. SCIM-III Mobility Categories and Stairs are recommended for assessing improvement in walking-related independence, and the SCI-FAI criterion for walking performance in everyday life.

## 1. Introduction

As a result of spinal cord injury (SCI), people can lose their independence in walking, which impacts their ability to function in their home and/or participate in the community, thus affecting their quality of life [1]. Achieving functional walking—defined as “the ability to walk with or without the aid of appropriate assistive devices, safely and sufficiently to carry out mobility-related activities of daily living”—is of high importance for patients with SCI [2].

Capacity refers to what a person can do in a clinical environment or test situation, while performance refers to what a person actually does in their habitual environment [3]. In rehabilitation of patients with SCI, various functional walking-capacity tests and disability-assessment scales (which typically assess mobility within the hospital, and related independence) are used [2,4]. Measures that assess walking performance/activity at home and in the community (e.g., through individuals’ self-reporting) are rare. It is not clear which measures and/or criteria are the most appropriate in terms of milestones for in-home or community functioning. At the same time, data are needed to assist in measuring change with walking tests (e.g., minimal detectable change (MDC) and minimal clinically important difference (MCID)) [5,6].

The Six-minute Walk Test (6MWT) is the most commonly used measure of walking capacity in rehabilitation [5,6]. To the best of our knowledge, its MCID for walking distance in patients with SCI has not yet been estimated. Reference equations [7] can be used to predict the distance covered in the 6MWT were the person healthy, taking into account their demographic data and physical activity.

Similar to walking distance, impairments of various physiological systems can also reduce walking speed. Comfortable walking speed measured over short distance is probably relevant for in-home activities in people with SCI [8]. Conversely, speed of fast walking over short distances is associated with crossing streets in elderly [9] and neurological patient populations [10,11], and thus with unrestricted walking in the community. The Ten-metre Walk Test (10MWT) is the most commonly used test in clinical practice for comfortable (10MWT_CS) and fast walking-speed (10MWT_FS) [5]. Only the MCID for 10MWT_FS was estimated in patients with SCI [12]. Cutoff values for the 6MWT and 10MWT_CS to distinguish between the ability to walk unlimited or limited in the community or at home only have been determined based on population daily walking activity using activity monitors in patients after stroke [13]. Normative values for comfortable walking speed were defined by a large meta-analysis [14] of data from apparently healthy adults which can also be used as criterion of assessing whether or not the patients’ walking ability improved with rehabilitation.

The aim of our retrospective study was to compare improvement-classification criteria of walking-ability and their concordance during first inpatient rehabilitation after SCI. We compared criteria to assess improvement in walking ability in terms of provisional milestones for in-home or community functioning and/or independence derived from outcome measures of walking capacity and walking performance. We were looking for universally applicable criteria regarding impairment aetiology, injury level and completeness, baseline walking status and rehabilitation duration.

## 2. Materials and Methods

This was a retrospective single-centre nation-wide cohort study. The University Rehabilitation Institute, Republic of Slovenia, is the only institution for primary rehabilitation of patients with SCI in the country. It defines 16-year-old patients as adults for rehabilitation purposes. Analysis of an institutional database was performed. At the time of analysis (years 2020–2023), the database contained data from 412 cases of patients with SCI admitted to first or repeated inpatient rehabilitation.

### 2.1. Participants

The data on all adults with a traumatic or non-traumatic SCI who were admitted to the first inpatient rehabilitation for at least 20 days over the period of four years were included in the analysis irrespective of the time since onset. The inclusion criteria were met by 281 participants. No participant fulfilling the inclusion criteria was excluded. They received a multidisciplinary, individually tailored rehabilitation programme with task-oriented physiotherapy 5 days/week, 1–2 h/day. This retrospective study complied with the Declaration of Helsinki and was approved by the Institutional Review Board (ID 035-1/2021-20/3.2), with the need for written informed consent waived.

### 2.2. Outcome Measures and Procedure

The analysis was performed for the outcomes of 6MWT, 10MWT, Walking Index for Spinal Cord Injury (WISCI-II), selected items of the Spinal Cord Independence Measure (SCIM-III) and the Functional Independence Measure (FIM), and the Spinal Cord Injury Functional Ambulation Inventory (SCI-FAI) Walking Mobility subscale at admission and discharge. These measures are regularly used in clinical practice at our facility.

The 6MWT was performed on a 60 m walkway with standardised encouragement, and the 10MWT was performed on a 14 m walkway. Two or three consecutive trials of comfortable walking were performed, followed by two trials of fast walking. Mean speed was calculated as the outcome of 10MWT_CS and 10MWT_FS. If the participant was unable to perform the tests with or without physical assistance, scores of 0 m (6MWT) and 0 m/s (10MWT) were recorded [5]. In patients with SCI, the 6MWT and the 10MWT have excellent intra-rater reliability, while their floor effects depend on the time since injury/onset [2,4,8,15].

Increase in 6MWT and both walking speeds (10MWT_CS and 10MWT_FS) from admission to discharge was calculated. For each participant, the proportion of expected walking distance was calculated using the reference equations for inactive men or women, taking into account age, height and weight [7]. The proportion of expected comfortable walking speed was calculated using normative values for gender and age-group [14]. The participants were categorised by externally derived provisional thresholds (derived from population after stroke) into Walking Ability Categories [13] according to their 6MWT and 10MWT_CS outcomes: no walking or non-functional walking (0 m; 0 m/s), in-home walking (≤204 m; ≤0.48 m/s), limited community walking (≤287 m; ≤0.92 m/s), or unlimited community walking (≥288 m; ≥0.93 m/s). Criteria of improvement for 6MWT and 10MWT_CS were increase by ≥MCID, by 20% [16], in % of expected value by 20 percentage-points, and in the Walking Ability Category. For the criteria of increase by at least 20% in 6MWT, 10MWT_CS and 10MWT_FS, any change from zero value was considered as meeting the criterion. In addition, the proportions of participants who improved their 10MWT_FS by 20% or to 0.9 m/s, 1.0 m/s, and 1.2 m/s [9], respectively, were calculated.

WISCI-II is a 21-level walking-capacity scale that integrates the level of physical assistance with the need for braces and walking aids for 10 m walk. It shows good responsiveness in patients with more severely impaired gait, while the 6MWT and the 10MWT_CS are superior for measuring improvements in patients with better walking ability [17]. WISCI-II has excellent intra-rater reliability, but a ceiling effect >3 months after injury/onset has been observed [4,17]. The criterion of improvement was a change from scores 0–5 (unable to walk or walk in parallel bars/non-functional walking) to ≥6 (various levels of walking) or from 6 to 19 to a higher score if this was walking without physical assistance (scores 9, 12, 13, 15, 16, 18, 19 or 20).

SCIM-III assesses independence in activities of daily living, including mobility. The second version of the Spinal Cord Independence Measure Indoor and Outdoor Mobility items showed medium to large responsiveness within first six months after injury [18]. First, SCIM-III Mobility Outdoors item-scores were categorised to community non-walkers (scores 0–3), community walkers with aids (scores 4–6) and independent community walkers (scores 7–8) [19]. Second, improvement in SCIM-III Stairs was defined as change between “unable to ascend or descend stairs” (score 0) to “ascend and descend with support or supervision” (score 1) or “without supervision” (scores 2–3). Third, scores of the SCIM-III Indoor Mobility (<10 m) and Outdoor Mobility (>100 m) were combined into five SCIM-III Mobility Categories, which were modified [20] as follows: wheelchair-dependent (indoor and Outdoor Mobility ≤ 2); supervised walking indoors and outdoors or wheelchair dependence outdoors (Indoor Mobility = 3; Outdoor Mobility ≤ 3); indoor walking and outdoor supervised walking or wheelchair (Indoor Mobility > 3; Outdoor Mobility ≤ 3); outdoor walking with aid (Indoor Mobility > 3; 3 < Outdoor Mobility ≤ 7); and walking without aid (indoor and Outdoor Mobility = 8).

FIM [21] assesses functional disability in a variety of domains. Improvements between FIM levels, from complete dependence to moderate dependence or to independence/no helper (score <3 to ≥3 or score <6 to ≥6), were assessed for FIM Walk (item L) and FIM Stairs (item M).

SCI-FAI [22] is a standardised outcome measure for assessing kinematics, assistive devices and walking mobility. It has excellent intra-rater reliability and a moderate ceiling effect [4,22]. The SCI-FAI Walking Mobility subscale was used to classify participants’ self-reported level of ambulation. The criterion of improvement was change between categories, from no walking/non-functional walking to in-home walking or to limited or independent community walking (score 0–1 to ≥2, score 2–3 to ≥4 or score 4 to 5).

### 2.3. Data Analysis

MDC_95_ and distribution-based change threshold (DBCT) as tentative estimates of MCID were calculated for 6MWT and 10MWT_CS. The intra-rater reliability coefficients (ICC 0.98 for both 10MWT_CS and 6MWT) from previous reliability studies [2,15] were used for MDC_95_ estimate. For DBCT the effect sizes were calculated from the SD at admission (medium effect-sizes (me): >0.5 SD; large effect-sizes (le): >0.8 SD) [23]. Agreement between all 23 criteria was assessed using Cohen’s kappa (<0.2 poor, 0.2–0.40 fair, 0.4–0.6 moderate, 0.6–0.8 substantial, >0.8 excellent agreement). For each variable, a pairwise comparison was performed between admission and discharge data using descriptive statistics. The data were analysed using IBM SPSS Statistics 31 (IBM Corp., Armonk, NY, USA, 2025).

The target for a preferable criterion was correspondence to transition to independent home or community walking combined with the ability to detect change beyond random variation.

## 3. Results

The study included 281 participants with SCI (Table 1). Their ages ranged from 16 to 86 years (interquartile range (IQR) 51–73 years) and the time since injury/disease diagnosis ranged from 0 to 60 months (median 2 months, IQR 1–4 months). The duration of rehabilitation ranged from 20 to 200 days (median 60 days, IQR 39–93 days). Some missing data occurred at admission and/or discharge, therefore corresponding sample sizes are listed when differing from 281 in Table 1, Table 2 and Table 3.

Descriptive statistics for walking abilities at admission and discharge are given in Table 2 and Table 3. At admission, 54% of participants scored in the lowest WISCI-II category, which dropped to 31% at discharge. However, according to the Walking Ability Categories, SCIM-III Mobility Categories and SCI-FAI Walking Mobility, 64–73% of the participants were unable to walk or their walk was non-functional on admission, and these proportions decreased to 31–42% at discharge (Table 3). From admission to discharge, 78, 67, and 63 participants transitioned from zero to a non-zero score on 6MWT, 10MWT_CS and 10MWT_FS, respectively. At discharge, 53% of participants reached/exceeded the estimated MDC of 6MWT (47 m) and 47% exceeded the MDC of 10MWT_CS (0.13 m/s).

The estimated DBCTme of the 6MWT was 69 m and the DBCTle was 111 m. The estimated DBCTme and DBCTle of the 10MWT_CS were 0.18 m/s and 0.29 m/s, respectively.

**Figure 1:**
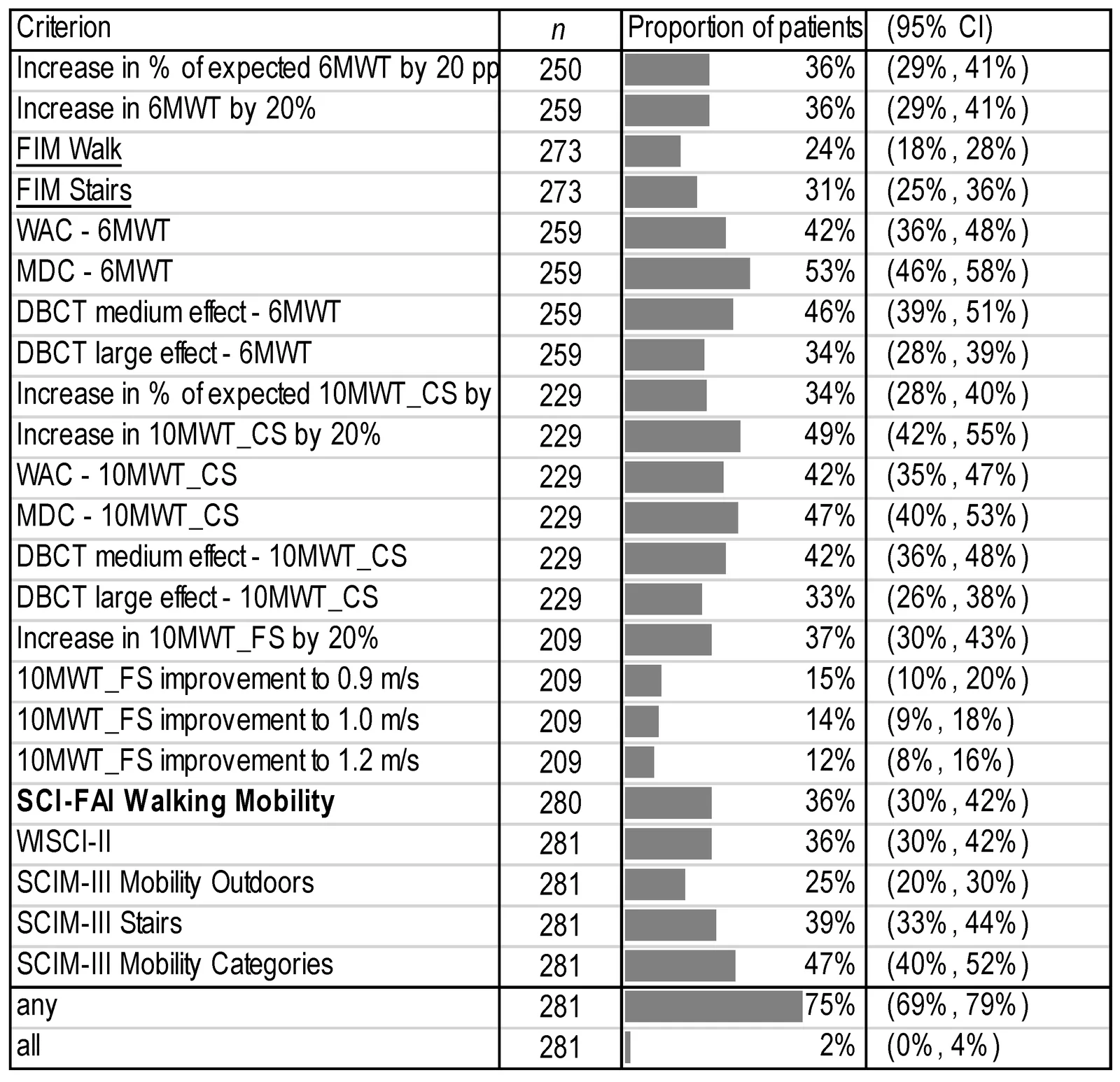
Proportion of patients who made progress according to a criterion (with 95% CI). Notes: CI—confidence interval; 6MWT—Six-minute Walk Test; FIM—Functional Independence Measure; WAC—Walking Ability Category; MDC—minimal detectable change; DBCT—distribution-based change threshold; 10MWT—Ten-metre Walk Test; CS—comfortable walking speed; FS—fast walking speed; SCI-FAI—Spinal Cord Injury Functional Ambulation Inventory; WISCI-II—Walking Index for Spinal Cord Injury; SCIM-III—Spinal Cord Independence Measure; underlined—disability scales; **bold**—performance/activity at home and in the community assessment scale.

The most frequently achieved criterion for improvement in walking ability was a 20% increase in 10MWT_CS, followed by improvement in SCIM-III Mobility Categories. The DBCTme of 6MWT was exceeded in 46% and the DBCTme of 10MWT_CS in 42% of participants. The same proportion of participants improved in the provisional Walking Ability Categories defined by 6MWT or 10MWT_CS criteria. The other criteria were in the following order: (5) SCIM-III Stairs; (6) increase in 10MWT_FS by 20%; (7) increase in 6MWT by 20%, increase in % of expected 6MWT by 20 percentage points, and improvement in WISCI-II and SCI-FAI Walking Mobility criteria; (8) DBCTle of 6MWT and increase in % of expected 10MWT_CS by 20 percentage points; (9) DBCTle of 10MWT_CS; (10) FIM Stairs dependence/independence levels; (11) SCIM-III Outdoor Mobility; (12): FIM Walk dependence/independence levels; and (13–15) improvement in 10MWT_FS to 0.9 m/s, to 1.0 m/s, to 1.2 m/s (Figure 1).

**Figure 2:**
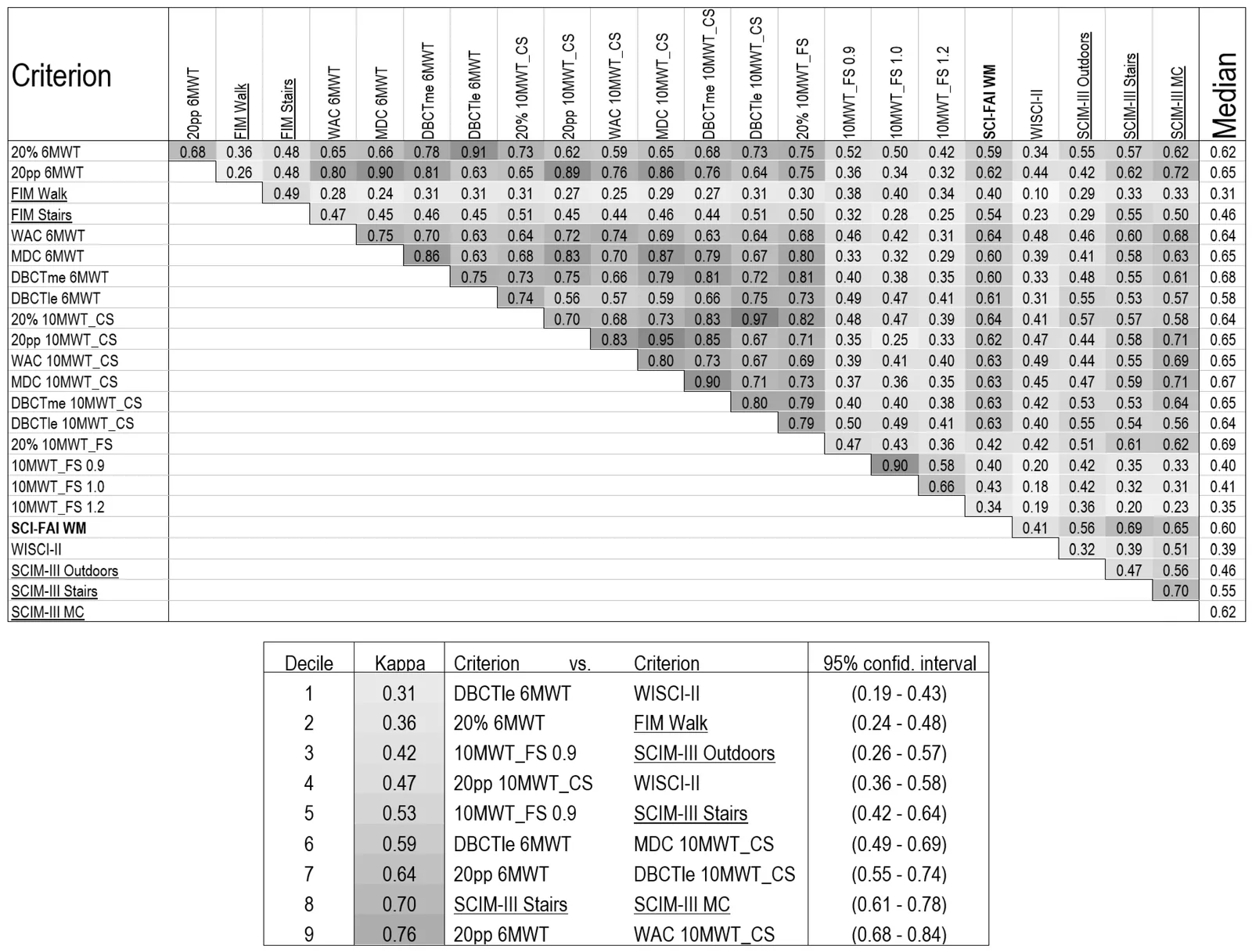
Agreement between criteria—Cohen’s *κ* (below the upper-diagonal matrix, a table of deciles of the observed distribution of *κ* values with confidence intervals is given for easier interpretation). Notes: 6MWT—Six-minute Walk Test; FIM—Functional Independence Measure; WAC—Walking Ability Category; MDC—minimal detectable change; DBCT—distribution-based change threshold; 10MWT—Ten-metre Walk Test; CS—comfortable walking speed; FS—fast walking speed; SCI-FAI WM—Spinal Cord Injury Functional Ambulation Inventory Walking Mobility; WISCI-II—Walking Index for Spinal Cord Injury; SCIM-III—Spinal Cord Independence Measure; underlined—disability scales; **bold**—performance/activity at home and in the community assessment scale; darker shading indicates higher *κ*.

Agreement between the criteria is presented in Figure 2. Agreement within and between the different 6MWT and 10MWT_CS criteria for improvement in walking ability was mostly substantial (for 47 pairs of criteria) or excellent (15 pairs). The 20% increase in 10MWT_FS was also in substantial or excellent agreement with the 6MWT and 10MWT_CS criteria, but the 10MWT_FS improvement to 0.9, 1.0 or 1.2 m/s showed moderate or fair agreement with these criteria. The WISCI-II improvement criterion showed only moderate or fair to poor agreement with all other walking-capacity improvement criteria (Figure 2).

Improvement in the SCIM-III Mobility Categories showed a substantial level of agreement with all 6MWT and 10MWT_CS improvement criteria, apart from both test’s DBCTle and the 20% increase in 10MWT_CS, for which agreement was moderate. Improvement in the SCIM-III Outdoor Mobility categories was moderately concordant with all criteria for improvement in walking capacity, except for improvement in 10MWT_FS to 1.2 m/s and the WISCI-II criterion, for which agreement was fair. SCIM-III Stairs improvement showed substantial agreement with the increase in % of expected 6MWT by 20 percentage-points and the 20% increase in 10MWT_FS. The SCIM-III Stairs criterion was moderately consistent with all other criteria of walking capacity except improvement in fast speed to 0.9–1.2 m/s (fair agreement) (Figure 2).

Improvement in FIM Walk dependence/independence levels agreed fairly with improvement in all walking-capacity criteria, as well as with all SCIM-III criteria. The exceptions were improvement of the 10MWT_FS to 1.0 m/s and of the SCI-FAI Walking Mobility criterion demonstrating moderate agreement with the FIM Walk criterion, and the WISCI-II criterion demonstrating poor agreement with it. Interestingly, the agreement between the improvement in FIM Stairs dependence/independence levels and most other criteria was moderate, except for improvement in speed to 0.9–1.2 m/s, WISCI-II and SCIM-III Outdoor Mobility (fair agreement) (Figure 2).

## 4. Discussion

Current walking-capacity assessment tools have been developed to document changes in outcomes that are sufficiently large to indicate clinically relevant improvements. The most important question is whether a patient is independent enough to return to their home environment [8]. To assist clinicians in interpreting outcomes, the MDC and MCID concepts have been developed [23], but they change with time or severity [24] and only few estimates exist for walking tests and disability scales specific to the SCI population. Milestones for functioning at home or in the community and associated independence may be more relevant for goal setting in physiotherapy and for assessing changes in improvement relevant to functioning of the individual. At the same time, we wanted to identify universally applicable criteria for classifying improvement regardless of various patients’ characteristics, because such criteria can serve for quality control, funding and/or institutional-comparison purposes.

Hence, in this retrospective study on a sample of 281 participants with SCI, we assessed improvement in walking ability using different criteria. Improvement in SCIM-III Mobility Categories was achieved in 47%. Estimated DBCT of 6MWT (69 m) was exceeded in 46%, and DBCT of 10MWT_CS in 42% of participants. Forty-two percent of participants improved in provisional Walking Ability Categories based on 6MWT or 10MWT_CS, 39% on SCIM-III-Stairs criterion, and 36% on the SCI-FAI criterion. In our study, 75% of participants improved on at least one criterion and only 2% on all the criteria. The latter indicates diversity of the studied criteria.

The majority of participants in our study were in the acute (median 2 months) or subacute rehabilitation stage and had incomplete impairment. It was shown that, especially in patients with incomplete SCI, neural plasticity is a main mechanism leading to greater functional recovery [25]. In addition, spontaneous neurological recovery has main influence in the acute rehabilitation stage. Therefore, a high proportion of improvement was expected.

The 20% increase in 10MWT_CS yielded the largest proportion of patients with improvement (49%). However, any change (i.e., from 0 m/s) was counted, which makes the clinical interpretation difficult, so the large proportion of improvement does not automatically speak in favour of this criterion. The estimated MDC of the 6MWT in our study (47 m) is similar to the value (45.8 m or a change by 22%) previously estimated [2] in patients with SCI. In our study, the estimated DBCT of the 6MWT was 69 m (for a medium effect) or 111 m (large effect), which is similar to MCID in the study with patients after stroke (all: 71 m) but larger than in their slow walking group (<0.4 m/s: 44 m) [6]. Our estimates for 10MWT_CS are 0.13, 0.18 and 0.29 m/s for MDC, DBCTme and DBCTle, respectively. The MDC confirms the previously estimated value in patients with SCI [2], and the DBCT is comparable to the MCID for 10MWT_FS (0.15 m/s) in this patient population [12]. In our study, more participants achieved MDC and DBCTme on 6MWT than on 10MWT_CS, but the proportions were similar for DBCTle. However, the criterion of 20% increase was achieved—in the order from highest to lowest proportion—on 10MWT_CS, 10MWT_FS, and 6MWT (49–36%). Physiotherapists tend not to test patients with poor walking ability with the 6MWT, especially at the beginning of rehabilitation, and therefore a discrepancy with 10MWT might exist [8], but this was not the case in our study.

Because the walking-capacity tests indicate the highest level of walking ability achieved by an individual patient in the clinical setting [2,3], it remains questionable whether these tests provide information about walking performance in the individual’s home and community environment [18]. One way to overcome this problem could be to use the Walking Ability Categories that were originally defined using activity-monitors in people after stroke [13]. According to these provisional criteria, in our study, the decrease in non-walkers and the increase in-home walkers at discharge were greater for 6MWT, but the increase in community walkers was higher for 10MWT_CS. There does not appear to be a clear hierarchy between improvement in 10MWT_CS and 6MWT criteria. Previously, a near-perfect correlation (*rho* = −0.95) was found between the two tests [26]. In patients with neurological deficits, both tests are limited by sensorimotor deficits and/or balance [8]. Because the importance of improving the gait pattern is emphasised, improvement in walking speed is more often addressed in training of initially high-functioning patients. This could have even greater impact on the improvement on the 10MWT_FS.

Increasing the percentage of expected 6MWT and 10MWT_CS by 20 percentage-points are probably less useful because additional calculations are required and their interpretation in terms of walking at home or in the community is not clear. The latter also applies to both DBCTle-based criteria. In addition, the criteria for increasing the 10MWT_FS to 0.9, 1.0 or 1.2 m/s appear to be the least useful for the general SCI population due to missing data and/or at least 65% of participants being unable to perform the test at fast speed according to the physiotherapist’s judgement at admission. Raising the 10MWT_FS to 1.0 m/s could be useful as an improvement criterion for high-functioning individuals.

The WISCI-II criterion for improvement was achieved by 36% of participants, but it had the lowest agreement with all other criteria of walking capacity and the SCI-FAI Mobility Scale (median *κ* = 0.39). In previous studies, WISCI-II outcomes showed moderate to high correlations with the 6MWT (*rho* = 0.60) [26], the 10MWT (*rho* = −0.68) [26] and the SCIM (*rho* = 0.70) [27]. The advantage of the WISCI-II is the assessment of patients with severe gait impairments and consequential walking limitations, including walking with physical assistance and consideration of the use of walking aids and braces. However, a previous retrospective study [8] showed that the order of its items is confusing, a strong non-linearity and redundancy of some items, while other items discriminated insufficiently. Independent walking should be rated better than walking dependent on the assistance of another person [26]. This was partly addressed in our WISCI-II improvement criterion. The discrepancy in the agreement between improvement criteria in our study is partly a consequence of the different scales (e.g., WISCI-II scores 6–8 for walking with physical assistance vs. the lowest category for this in other scales). In addition, as expected, criteria derived from the same walking test showed relatively high agreement among them.

There is low-quality evidence that general exercise or robot-assisted gait training increase total SCIM-III or FIM scores [28]. Participants in our study received these therapeutic procedures, but we only focused on the improvement of gait-related items. Except for SCIM-III Mobility Outdoors, where supervision was accounted for in the first category (score 3—no improvement), improvement for the other SCIM-III and FIM criteria was defined as change in dependence on another person. Improvement was the most frequent on the SCIM-III Mobility Categories (47%) and, as expected, the rarest on SCIM-III Mobility Outdoors (25%) and FIM Walk (24%). The SCIM-III Mobility Categories show a substantial level of agreement with other criteria (median *κ* = 0.62), and SCIM-III Stairs has moderate agreement with other criteria (median *κ* = 0.55) and greater improvement rate (39%) than FIM Stairs. FIM Walk and FIM Stairs have fair (median *κ* = 0.31) and moderate (median *κ* = 0.46) agreement with other criteria, respectively. In agreement with a Rasch-model-based comparison of SCIM-III and FIM [29], which supported the advantage of using SCIM-III to assess functional independence of patients with SCI in rehabilitation, we recommend the use of SCIM-III Mobility Categories and SCIM-III Stairs to assess improvement in walking-related independence. The SCIM-III Mobility Outdoors shows moderate agreement with other criteria (median *κ* = 0.46), but because of excessive strictness for the general target-population it could be useful as an improvement criterion for high-functioning individuals.

Due to limitations in assessing walking capacity, it has been recommended to additionally assess walking performance, which depends not only on a person’s abilities and motivation but also on the limitations imposed by the environment [2]. In our study, improvement in the SCI-FAI Walking Mobility subscale was observed in 36% of participants and showed moderate agreement with other criteria (median *κ* = 0.60). SCI-FAI Walking Mobility subscale is a self-reported/clinician-administered estimate of walking performance which has limitations of recall, social desirability and uncertain exposure to home and community environments in inpatients. However, the inpatients at our institution whenever possible, spend at least one weekend at home before being discharged from rehabilitation in order to experience functioning at their home environment, which may help with more realistic estimates of walking performance. A longitudinal observational study [24] reported that after outpatient physiotherapy, walking capacity (10MWT_CS) improved in all patients after stroke, but walking performance (measured with activity monitors) improved in none of them; whereas, 9% of patients with Parkinson disease who improved in walking capacity also improved in walking performance. In our study, 42% of participants exceeded the DBCTme for 10MWT_CS, and 36% improved on the SCI-FAI Walking Mobility criterion (*κ* = 0.63). Hence, SCI-FAI Walking Mobility criterion provides complementary self-reported information on walking performance and its improvement if activity monitors are not available.

### Study Limitations

The retrospective nature of the study might be considered a limitation primarily because of increased likelihood of incomplete data; however, it helped reducing observer bias during assessment. Participants with complete tetraplegia (4%) were not excluded from the study, but this can also be seen as an advantage because the study results can thus be interpreted for the general population with SCI at their first inpatient rehabilitation. Although use of different walking aids [30] and balance deficits affect walking-ability outcomes, this was not systematically addressed in our study. In addition, the thresholds for 10MWT_CS and 6MWT used to derive the Walking Ability Categories, which imply improvement in real-life walking performance, were determined in patients post stroke [13]. However, these thresholds are likely to be universal from the functioning point of view, such as the walking speed for in-home walking or crossing a street, and walking distance needed for limited community walking. Nevertheless, because pathophysiological consequences of SCI on walking and effects of assistive devices differ from stroke population, prospective validation of these criteria in the SCI population is needed.

## 5. Conclusions

Although several criteria classified similar proportions of patients as improved, multidimensional assessment is recommended. As expected, criteria derived from the same walking test showed relatively high agreement. The estimated DBCT corresponding to 0.5 SD and the Walking Ability Categories for both the 6MWT and the 10MWT_CS might be potentially useful to assess improvement in walking capacity after inpatient rehabilitation in patients with SCI. However, they require prospective validation against patient-centred real-world mobility anchors, including activity monitors in the SCI population.

SCIM-III Mobility Categories and SCIM-III Stairs may offer clinically interpretable information about improvement in walking-related independence. SCI-FAI Walking Mobility subscale provides complementary self-reported information on walking performance in daily life.

To summarise, the selected set of broad-scope criteria for classifying improvement in walking ability during inpatient rehabilitation after SCI may serve for setting physiotherapy goals and assessing their achievement, guiding future research, and hospital-management purposes.

## Figures and Tables

**Table 1:** Demographics and clinical characteristics of participants (*n* = 281), and their use of walking aids.

Characteristic	Value
Gender, % men	66.5
Age (years), mean (SD)	61 (15)
Duration of hospitalisation (days), mean (SD)	69 (38)
Time since onset (*n* = 277) (months), mean (SD)	4.8 (15.7)
Non-traumatic/traumatic injury (*n* = 278) (*n*)	154/124
Paraplegia, unspecified (*n*), non-traumatic/traumatic	6/6
Paraplegia, incomplete (*n*), non-traumatic/traumatic	86/36
Paraplegia, complete (*n*), non-traumatic/traumatic	9/13
Tetraplegia, unspecified (*n*), non-traumatic/traumatic	3/5
Tetraplegia, incomplete C1–4 (*n*), non-traumatic/traumatic	19/26
Tetraplegia, incomplete C5–8 (*n*), non-traumatic/traumatic	26/32
Tetraplegia, complete C1–4 (*n*), non-traumatic/traumatic	2/1
Tetraplegia, complete C5–8 (*n*), non-traumatic/traumatic	3/5
Walking aids, admission (*n* = 130)/discharge (*n* = 194) (*n*)	
Walker	11/0
Rollator	72/107
Forearm crutches	19/27
Walking sticks	7/20
None	21/40

**Table 2:** Descriptive statistics of the walking capacity tests outcomes and their derivates at admission and discharge, and change from admission to discharge.

Walking Capacity Test Outcomes and Their Derivates	Admission		Discharge		Change		
	Mean (SD)	Median (Range)	Mean (SD)	Median (Range)	Mean (SD)	Median (Range)	No. of Negative
6MWT (m) (*n*_A_ = 269, *n*_D_ = 265, *n*_C_ = 259)	84 (138)	0 (0–575)	173 (179)	135 (0–740)	90 (110)	55 (−63–566)	7
% of expected 6MWT (*n*_A_ = 259, *n*_D_ = 255, *n*_C_ = 250)	15 (24)	0 (0–94)	31 (31)	26 (0–121)	16 (19)	11 (−10–82)	5
10MWT_CS (m/s) (*n*_A_ = 257, *n*_D_ = 239, *n*_C_ = 229)	0.2 (0.4)	0 (0–1.6)	0.4 (0.5)	0.3 (0.0–0.7)	0.2 (0.3)	0.1 (−0.5–1.6)	12
% of expected 10MWT_CS (*n*_A_ = 257, *n*_D_ = 239, *n*_C_ = 229)	17 (28)	0 (0–120)	35 (36)	29 (0–127)	17 (24)	7 (−36–121)	12
10MWT_FS (m/s) (*n*_A_ = 241, *n*_D_ = 226, *n*_C_ = 209)	0.3 (0.5)	0.0 (0.0–1.9)	0.6 (0.7)	0.3 (0–2.1)	0.3 (0.4)	0 (−0.3–2.0)	4
WISCI-II (*n*_A_ = 281, *n*_D_ = 281, *n*_C_ = 281)	6 (7)	0 (0–20)	10 (7)	12 (0–20)	4 (5)	1 (−11–20)	11

*n*_A_—no. of patients tested at admission; *n*_D_—no. of patients tested at discharge; *n*_C_—no. of patients tested at admission and discharge; 6MWT—Six-minute Walk Test; 10MWT_CS—Ten-metre Walk Test with comfortable walking speed; 10MWT_FS—Ten-metre Walk Test with fast walking speed; WISCI-II—Walking Index for Spinal Cord Injury.

**Table 3:** Descriptive statistics of categorical variables analysed as criteria for improvement of walking ability.

Categories/Scales	Scores (Adjusted)	Admission		Discharge	
		No.	%	No.	%
Provisional WAC—6MWT (*n*_A_ = 269, *n*_D_ = 265)	0 m: NW or NFW	172	64%	93	35%
	≤204 m: In-home walking	47	18%	71	27%
	≤287 m: Limited CW	16	6%	28	11%
	≥288 m: Unlimited CW	34	13%	73	28%
Provisional WAC—10MWT_CS (*n*_A_ = 257, *n*_D_ = 240)	0 m/s: NW or NFW	168	65%	90	38%
	≤0.48 m/s: In-home walking	41	16%	48	20%
	≤0.92 m/s: Limited CW	32	13%	55	23%
	≥0.93 m/s: Unlimited CW	16	6%	47	20%
SCIM-III Mobility Outdoors (*n*_A_ = 281, *n*_D_ = 281)	0–3: Community NW	224	80%	165	59%
	4–6: CW with walking aids	35	13%	79	28%
	7–8: Independent CW	22	8%	37	13%
SCIM-III Stairs (*n*_A_ = 281, *n*_D_ = 281)	0: Unable to ascend or descend stairs—NW stairs	198	71%	112	40%
	1: Ascend and descend at least 3 stairs with support or supervision	26	9%	18	6%
	2–3: Ascend and descend at least 3 stairs without supervision	57	20%	151	54%
SCIM-III Mobility Categories (*n*_A_ = 281, *n*_D_ = 281)	1: Wheelchair-dependent—NW	183	65%	97	35%
	2: Supervised walking indoors and outdoors or wheelchair dependence outdoors	32	11%	36	13%
	3: Indoor walking, outdoor supervised walking/wheelchair	9	3%	32	11%
	4: Outdoor/CW walking with aid	36	13%	80	29%
	5: Outdoor/CW walking without aid	21	8%	36	13%
FIM Walk (*n*_A_ = 278, *n*_D_ = 276)	0: Wheelchair dependence—NW	128	46%	128	46%
	1–2: Complete dependence—NW	39	14%	4	1%
	3–5: Moderate dependence (in-home walking)	48	18%	31	11%
	6: Independence; min. 50 m, with assistive devices, slow	45	16%	73	26%
	7: Independence; safe, fast, min. 50 m, without assistive devices	18	7%	40	15%
FIM Stairs (*n*_A_ = 278, *n*_D_ = 276)	1–2: Complete dependence—NW stairs	201	72%	125	45%
	3–5: Moderate dependence	24	9%	57	21%
	6: Independence; ascend and descend 1 floor, stair handle, assistive devices or slow, risk of falling	45	16%	67	24%
	7: Independence; ascend and descend 1 floor, without support, safe, fast	8	3%	27	10%
**SCI-FAI Walking Mobility** (*n*_A_ = 281, *n*_D_ = 280)	0–1: NW or NFW (for exercise only)	205	73%	116	42%
	2–3: In-home walking	17	7%	41	15%
	4: Limited CW	9	3%	29	10%
	5: Independent CW	50	18%	94	34%

*n*_A_—no. of patients assessed at admission; *n*_D_—no. of patients assessed at discharge; 6MWT—Six-minute Walk Test; 10MWT_CS—Ten-metre Walk Test with comfortable walking speed; WAC—Walking Ability Category; SCIM-III—Spinal Cord Independence Measure; FIM—Functional Independence Measure; SCI-FAI—Spinal Cord Injury Functional Ambulation Inventory; underlined—disability scales; **bold**—performance/activity at home and in the community scale; NW—non-walking; NFW—non-functional walking; CW—community walking; darker shading indicates higher category.

## Data Availability

The original contributions presented in this study are included in the article/Supplementary Materials. Further inquiries can be directed to the corresponding author.

## Supplementary Materials

The following supporting information can be downloaded at: https://www.mdpi.com/article/10.3390/healthcare14162668/s1, File S1: Raw data S1; File S2: No. of participants with assigned zero score, missing assessments and change from zero to a non-zero score on the walking capacity tests.

## Abbreviations

The following abbreviations are used in this manuscript:

SCI	Spinal cord injury
MDC	Minimal detectable change
MCID	Minimal clinically important difference
6MWT	Six-minute Walk Test
10MWT	Ten-metre Walk Test
10MWT_CS	Ten-metre Walk Test for comfortable walking-speed
10MWT_FS	Ten-metre Walk Test for fast walking-speed
WISCI-II	Walking Index for Spinal Cord Injury
SCIM-III	Spinal Cord Independence Measure
FIM	Functional Independence Measure
SCI-FAI	Spinal Cord Injury Functional Ambulation Inventory
DBCT	distribution-based change threshold
DBCTme	Minimal clinically important difference estimated for medium effect-size
DBCTle	Minimal clinically important difference estimated for large effect-size
