# Supplementary material for: Comparison and Agreement of Criteria for Classifying Improvement in Walking Ability During Inpatient Rehabilitation After Spinal Cord Injury
Source: Healthcare (Basel). 2026 Aug 21;14(16):2668. doi: 10.3390/healthcare14162668 (PMC13511803; doi:10.3390/healthcare14162668)
Supplement: Supplementary file 1 [file healthcare-14-02668-s001.zip › S2_No. of participants with assigned zero score, missing assessments and change.pdf]

Comparison and Agreement of Criteria for Classifying Improvement in Walking Ability During Inpatient Rehabilitation After Spinal Cord Injury

Urška Puh [1,2,*], Tea Čuk [1], Bojan Čeru [1], Gaj Vidmar [1,3,4]

[1] University Rehabilitation Institute, Republic of Slovenia, 1000 Ljubljana, Slovenia; tea.cuk@ir-rs.si (T.Č.); bojan.ceru@ir-rs.si (B.Č.); gaj.vidmar@ir-rs.si (G.V.)
[2] Faculty of Health Sciences, University of Ljubljana, 1000 Ljubljana, Slovenia
[3] Faculty of Medicine, University of Ljubljana, 1000 Ljubljana, Slovenia
[4] Faculty of Mathematics, Natural Sciences and Information Technologies, University of Primorska, 6000 Koper, Slovenia
*Correspondence: urska.puh@ir-rs.si

Table S2. Number of participants with assigned zero score, missing assessments and change from zero to a non-zero score on the walking capacity tests

Walking capacity test	Assigned zero because of physical assistance (*n*)		Unable to attempt the test for medical or safety reasons (*n*)		Genuinely missing assessments (*n*)		Transitioned from zero to a non-zero score (Change)
	Admission	Discharge	Admission	Discharge	Admission	Discharge	*n*
6MWT	19	5	153	88	12	16	78 (45%)
10MWT_CS	15	3	153	87	24	41	67 (40%)
10MWT_FS	7	2	175	106	39	54	63 (35%)

6MWT – Six-minute Walk Test, 10MWT_CS – Ten-meter Walk Test with comfortable walking speed, 10MWT_FS – Ten-meter Walk Test with fast walking speed.
